# Primary retroperitoneal mucinous cystadenoma of borderline malignancy in a male patient. Case report and review of the literature

**DOI:** 10.1186/1477-7819-9-98

**Published:** 2011-08-27

**Authors:** Evangelos Falidas, Stefanos Konstandoudakis, Konstantinos Vlachos, Fotios Archontovasilis, Stavros Mathioulakis, Stavros Boutzouvis, Constantinos Villias

**Affiliations:** 1 1st Department of General Surgery, 417 NIMTS, Veterans Hospital of Athens, Monis Petraki 10-12, Athens, 11521, Greece; 2 Department of Pathology, 417 NIMTS, Veterans Hospital of Athens, Monis Petraki 10-12, Athens,11521, Greece; 3 Department of Therapeutic Endoscopy and Laparoscopic Surgery, 'Iaso' General Hospital, Mesogion Avenue 264, Athens,15562, Greece

## Abstract

**Background:**

Primary retroperitoneal mucinous cystadenoma of borderline malignancy represents a rare tumor, with unclear histogenesis, concerning almost exclusively women. Only two cases concerning male patients have been reported.

**Case report:**

We herein report a case of a 37 year old man undergone laparotomy for a sizable retroperitoneal tumor resulting after the histological examination to a primary retroperitoneal mucinous cystadenoma of borderline malignancy.

**Conclusion:**

This is the third case of primary retroperitoneal mucinous cystadenoma of borderline malignancy in a male patient reported in the literature. The preoperative diagnosis is impossible. Laparotomy constitutes the only diagnostic and curative approach.

## Background

The primary retroperitoneal mucinous cystadenoma is a rare tumor that affects, almost exclusively women [1]. The preoperative diagnosis is impossible in most of cases because of the rarity, the position and the texture of the tumor. We report a case of a 37 year-old male patient, with primary retroperitoneal mucinous cystadenoma of borderline malignancy; this may be the third case registered in the literature.

### Case report

A 37 year old man arrived at the outpatient facilities of our clinic complaining of a remittent pain at the right lateral abdomen and a palpable mass that was constantly growing up during the last twelve months. His medical history included an uneventful appendicectomy in his childhood, 27 years ago and spontaneous pneumothorax 15 years ago. By that time, he was under medication with antacids because of gastritis. He did not mention any evacuation or urination symptoms.

**Figure 1:**
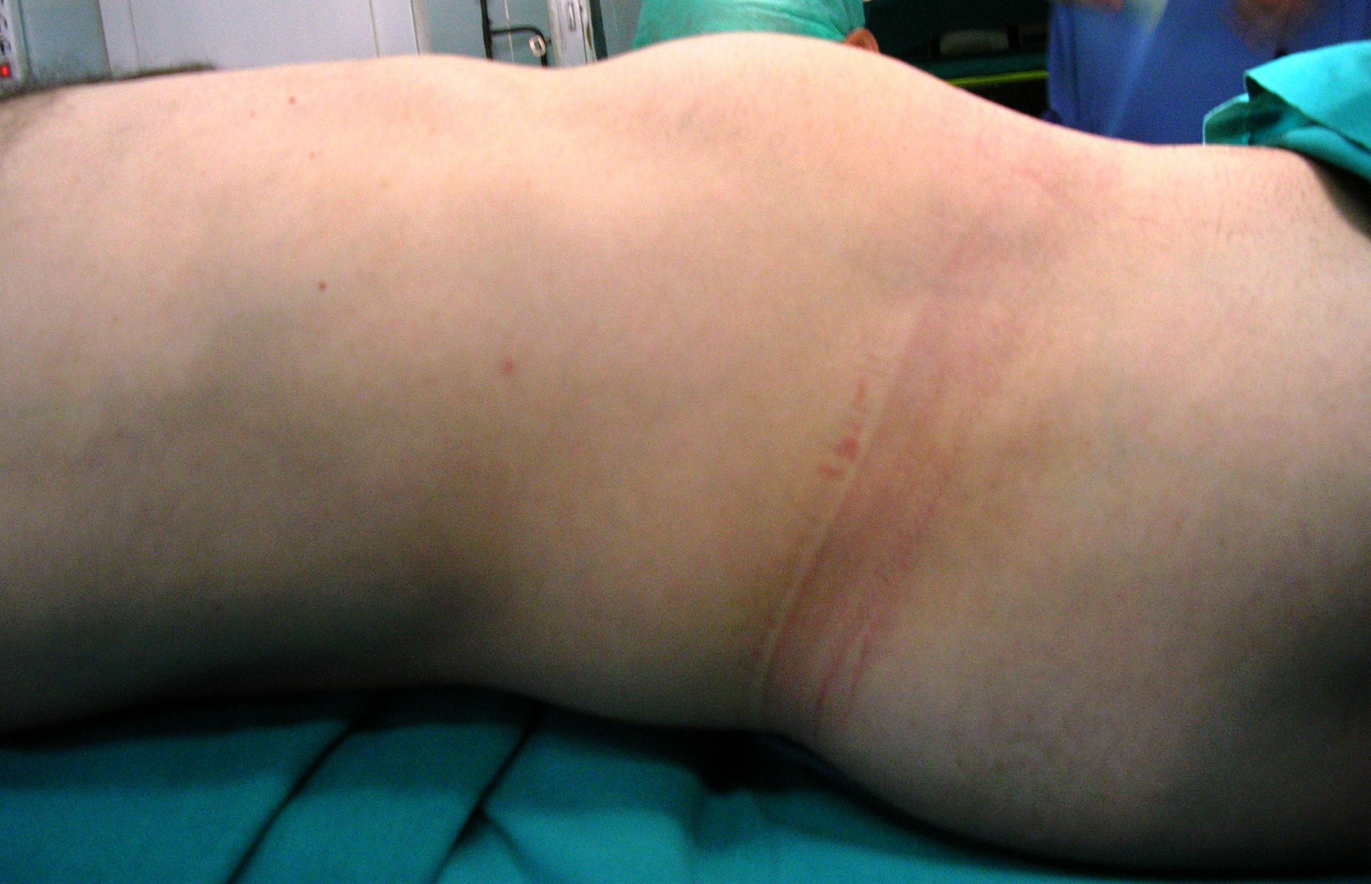
**Deformation of the right abdomen due to sizable retroperitoneal mass in a 37-year old man**.

**Figure 2:**
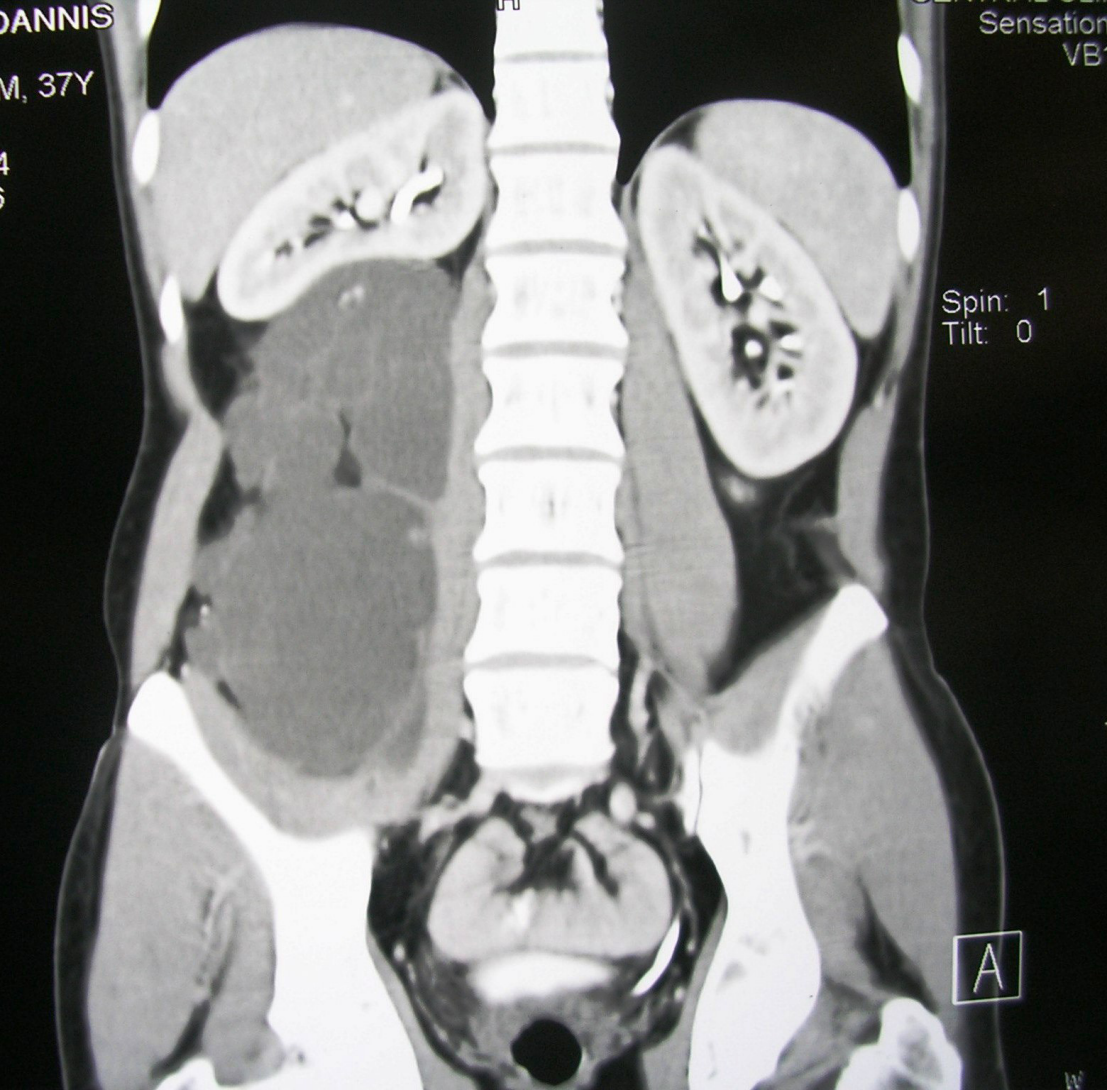
**Coronal CT image describing the size of the tumor and its mass effect to the right kidney**.

**Figure 3:**
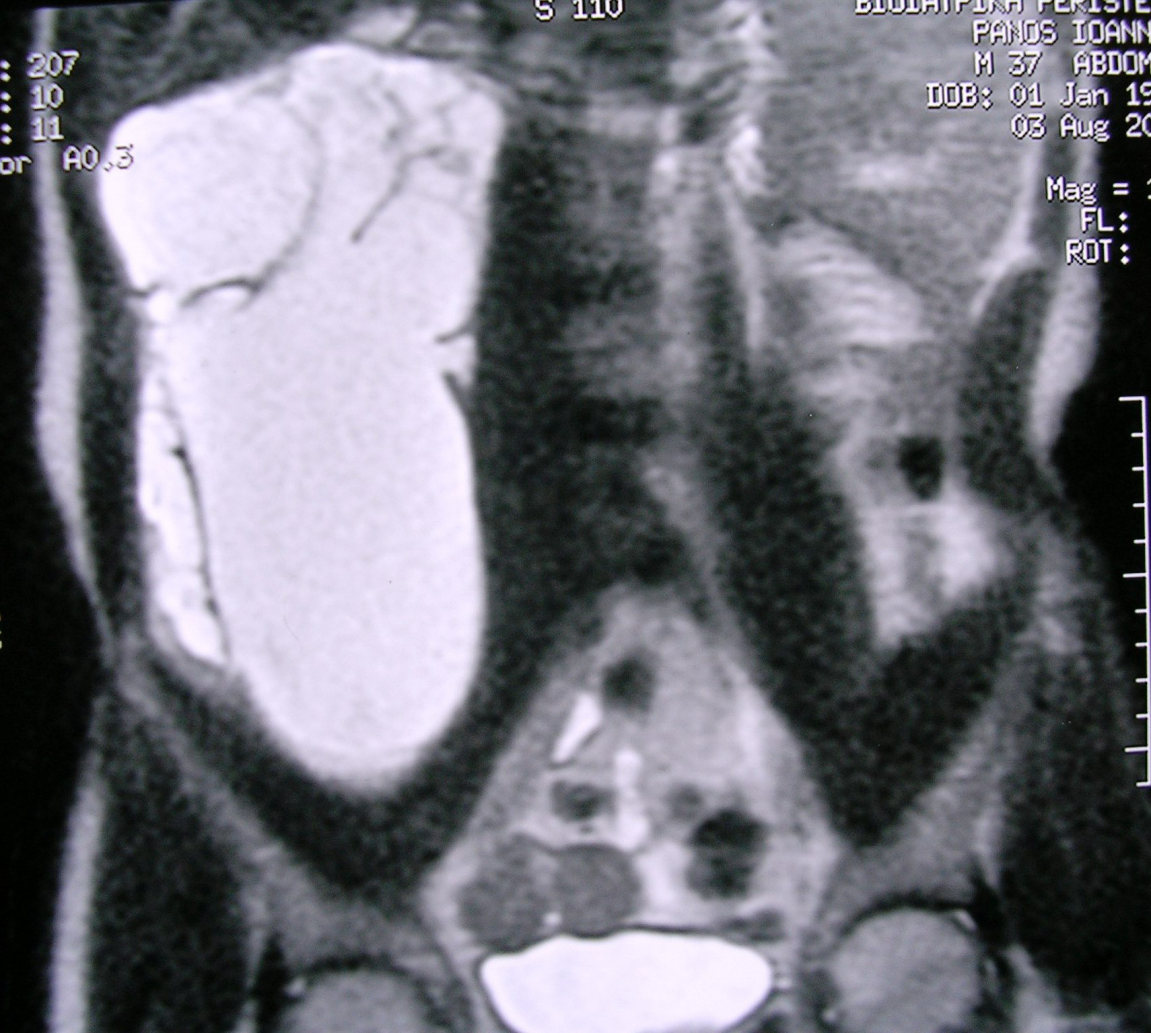
**T2 coronal MR image without fat suppression, demonstrating the cystic component of the lesion with internal septa**.

**Figure 4:**
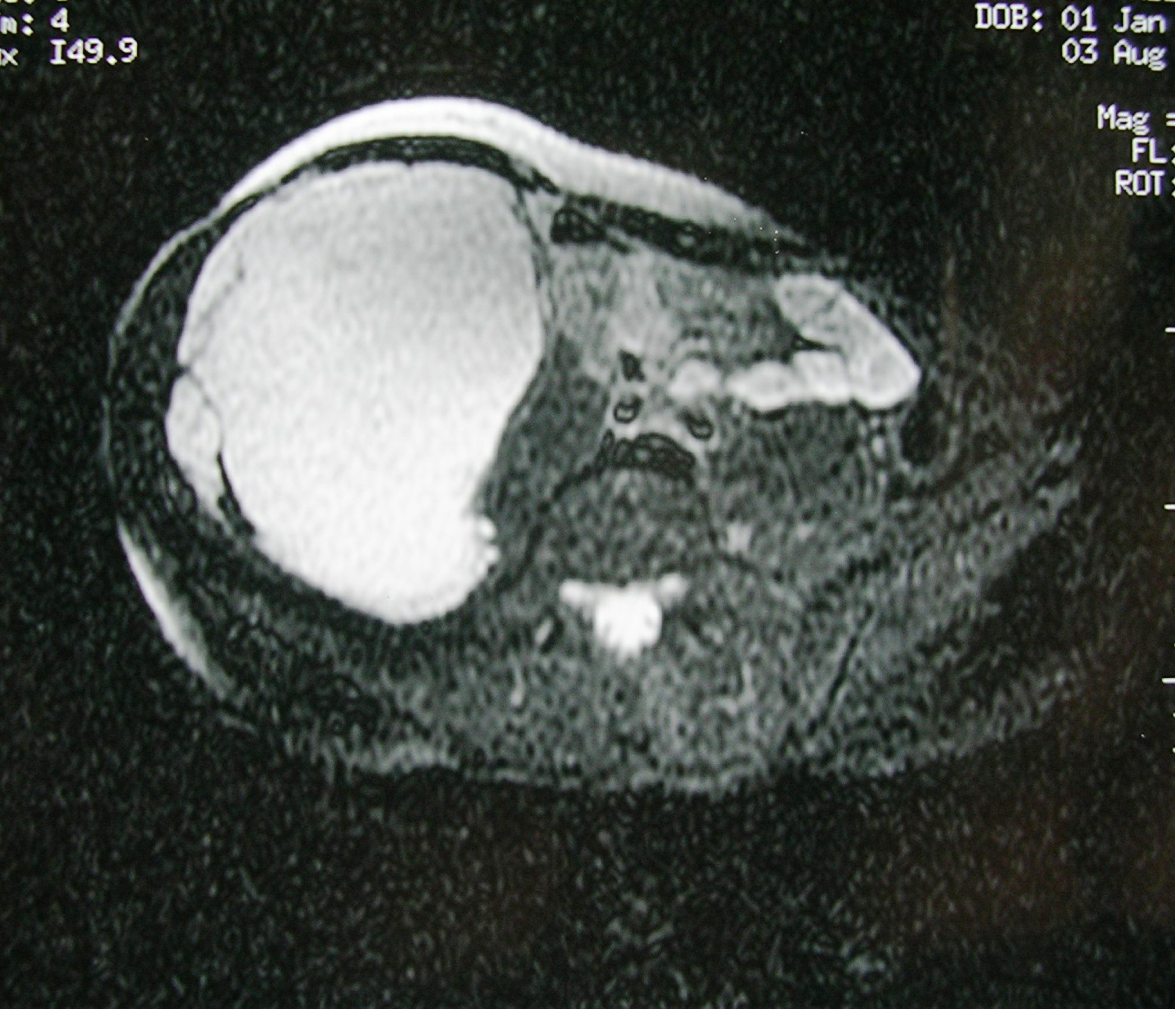
**Axial T2 MR image with fat suppression revealing the cystic character of the tumor**.

Physical examination revealed a firm, sizeable and hard mass at the right abdomen (Figure 1), extending from the inferior ribs to the right iliac crest. Laboratory findings were within normal limits. The ultrasound (US) of the abdomen demonstrated a compact mass of uneven shape at the right abdomen, extended from the liver to the minor pelvis. Abdominal computed tomography (CT) scan, demonstrated a cystic textured lesion, extended from the lower pole of the right kidney to the right iliac fossa, with a cefalocaudal diameter of 11 cm (Figure 2). There were small diaphragms within the mass, enforced by the intravenous administration of a contrast essence. The tumor was expelling not only the homolateral ureter but also the small and the large intestine to the left, however, without any findings of obstruction. The magnetic resonance imaging (MRI) of the abdomen demonstrated a cystic tumor with diaphragms (Figure 3, Figure 4), extending along the right abdomen from the inferior pole of the right kidney to the right iliac crest. The tumor measured a maximum cefalocaudal diameter of 22 × 10 cm, causing a slight pressure of the inferior vena cava. The administration of paramagnetic essence revealed an uneven peripheral enhancement of signaling. Three focal lesions of magnetic abnormal signaling were brought out at the liver segments II, VI, VII, and VIII, suggesting secondary metastasis or hemangiomas. Colonoscopy and upper GI endoscopy was carried out in order to exclude involvement of the gastrointestinal tract. Gastritis was the only abnormal finding.

The patient underwent laparotomy. A retroperitoneal cystic mass of gelatinous-like content, arising from the paravertebral space of the L_1_-L_2_-L_3 _vertebra, was confirmed. An "en block" resection of the tumor was performed. The small and the large intestine as well as the right ureter did not present signs of primary or metastatic involvement.

The first postoperative hours, the patient complained of sub-hyperesthesia at the inner surface of the thigh that was extended to the middle of the calf. In addition, he asserted a reduction of muscle power at his crur (thigh flexion debility). Within the next 24 hours, he underwent an electromyogram which demonstrated a possible neurogenic damage, due to lesions of the L_2_-L_3 _level. Methylprednisolone (250 mg) was administrated as a single boost dose, followed by dexametasone (8 mg three times a day). The patient was discharged the fourth postoperative day without any other postoperative complication.

**Figure 5:**
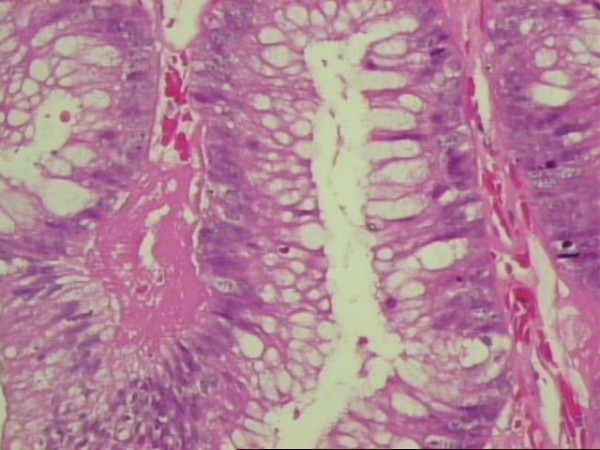
**Hematoxylin and eosin stain demonstrating papillary formations lined by columnar cells producing mucin, with moderate cytological atypia (original magnification ×40)**.

**Figure 6:**
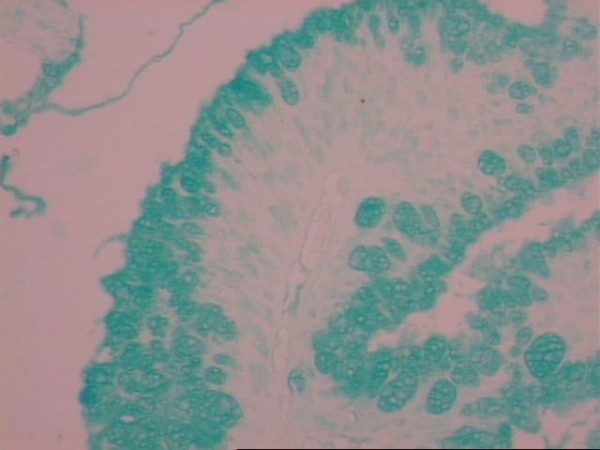
**Alcian Blue stain revealing the presence of intracytoplasmatic mucin (original magnification ×40)**.

**Figure 7:**
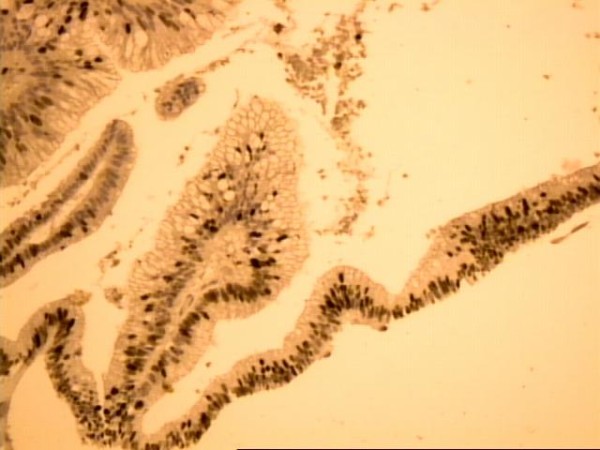
**Ki-67 stain revealing positivity of the nuclei of the epithelial lining cells ranged from 30 to 50% (original magnification ×20)**.

The tumor weighed 957 g and measured 22 × 14 × 4,5 cm. The histological examination described internal cysts within the mass, measuring from 1 to 4 cm in diameter. The thickness of the cystic wall was variable (from 0,1 to 0,5 cm). Microscopically the inner surface of the cystic wall revealed filiform and branching papillae lined by mucin-containing atypical epithelial cells (Figure 5). The lining cells were stratified, generally to two or three layers, and the nuclear atypia was mild to moderate. The collagen fibers of the wall were disintegrated by pools of mucous, epithelial cells and calcifications. Lymphocytic infiltration was also observed. The PAS-D and Alcian Blue stain was positive as well as the immunihistochemical stain for keratine 8/18, keratine 20, pankeratine, CEA and Ki-67 (Figure 6, Figure 7).

Two months later the patient had a new abdominal CT-scan which demonstrated absence of residual disease. The electromyogram was compatible with a chronic neurogenic damage of the quadriceps muscle. The next two abdominal CT-scans, performed 6 and 12 months later, showed no recurrence. A significant clinical improvement was observed in patient's neurological status, followed by a considerable increase of muscle power.

## Discussion

Primary retroperitoneal tumors are rare (0,01-0,2% of all neoplasias) [2,3]. Most of them are malignant with characters of a non specific symptomatology. Delayed diagnosis of these tumors is common. The most frequent histological types are fibrosarcomas and liposarcomas. On the other hand, leiomyofibromas, leiomyosarcomas, malignant fibrous histiocytomas, neurofibromas, and rabdomyosarcomas are less common [2,3]. Primary retroperitoneal mucinous cystadenomas (PRMC), mucinous cystadenomas of borderline malignancy (PRMC-BM) and cystadenocarcinomas (PRMC-C) are extremely rare tumors concerning almost exclusively women [1].

Baker et al [1] reviewing the literature from 1966 up to 2006 identified 45 cases of PRMC and 25 cases of PRMC-C. In the same study they reported 9 cases of PRMC-BM, only one case concerned a male patient. In 2009, Roma et al [4] in a retrospective analysis of 18 retroperitoneal mucinous tumors, identified 7 cases of PRMC-BM. In this study all patients were women. Lai et al [5] reported the first case of pure PRMC in a male patient. Motoyama et al [6] reported the first case of PRMC-BM in a male patient. Two similar cases of PRMC and PRMC-BM were reported in 2008 and 2009 respectively [7,8]. This paper presents the third case of primary retroperitoneal mucinous cystadenoma of borderline malignancy in a male patient.

Tumors of epithelial derivation located in the retroperitoneum are rare because of the non existence of epithelial cells in this area. Nevertheless, Roth et al [9] reported Müllerian type epithelium tumors. PRMC and PRMC-C present histological and morphological characteristics similar to the mucinous tumors of the ovaries (ovarian-like stroma) [10]. This fact further supports the theory that tumor's growth is due to an ectopic or aberrant ovarian tissue. However, ovarian tissue has been rarely detected in histological speciments and in conjunction to the fact that these tumors appear in men weakens the theory. Gotoh et al [11] asserted that the peritoneal epithelium possesses the potential of Müllerian differentiation as it appears in almost all the ovarian tumors. The overgrowing of the mucinous epithelium on teratoma or genitourinary remnants constitutes two other theories of this context [12]. However, most of the authors agree that mucinous tumors originate from multipotential mesothelial cells, entrapped in the retroperitoneum during the growing process. These cells undergo a mucinous metaplasia, creating cystic mucinous inclusions with cytological changes and malignant phenotypes [13,14].

The preoperative diagnosis is difficult because of the non specific presenting symptoms and the vague preoperative imaging tests. The mucinous cystadenoma is usually asymptomatic. Abdominal discomfort or sickness, distension or pain and rarely intermittent intestinal occlusion are the most common reported symptoms [2,3,13,14]. Acute abdomen due to intestinal obstruction caused by retroperitoneal mucinous cystadenoma has also been reported [15].

Preoperative studies with US, CT and MRI identify abdominal masses, often reveal the nature of the tumors, but insufficient to lead to a definite diagnosis. Matsubaras et al [13] in a review effectuated from 1966 to 2005 found only two reports concerning the preoperative "suspicion" of a retroperitoneal cystic tumor. Others, like Thamboo [16] consider that the CT examination lays the suspicion of a retroperitoneal tumor, provided other elements are seriously considered like the displacement of the ureter, kidneys, and large vessels or intestine.

The abdominal US is not specific [5], may reveal the unilobular or multilobular tumor's character but is unable to determine its origin and extension. The diagnostic value of CT and MRI is similar. CT scan, although easily accessible, exposes the patient in irradiation and does not offer an adequate description of the tumor's relation with the soft tissues [5]. However, CT reveals the extension of the mass and shows better mural calcifications. The later, are considered to be important findings in the differential diagnosis between cystic teratoma and cystadenoma. Calcifications found within the mass support the diagnosis of teratoma while mural calcifications enforce the suspicion of cystadenoma [17]. On the other hand, MRI offers a rich variety of images and reveals better the correlations between the mass and the soft tissues. MRI seems to be more specific in describing the extension of the mass in the pelvis. The various scans allow the surgeon to determine possible associations between the mass and the pancreas, the kidneys and the ovaries, organs that could be potentially implicated in similar cystic lesions [5,13,16]. In our case, the tumor was recognized by US and its extension and association with the neighboring organs was estimated by CT and MRI. Lymphangiomas, cystic teratomas, lymphoceles, urinomas and cystic mesotheliomas were also included in the differential diagnosis. The tumor markers do not seem to have any specificity neither at PRMC-BM nor at PRMC-C [12,14]. In our case, tumor markers (CA-125, CA-19.9, and CEA) were within normal limits. Fine Needle Aspiration (FNA) under the guidance of US or CT is rarely diagnostic because the taken material mainly represents the core of the tumor, which is mostly consisted of mucus, thus unable to demonstrate histopathological characteristics [6].

Although the laparotomy has a double role, diagnostic as well as curative, the approach may be different in accordance to sex. There is a general agreement that a radical resection should be performed in both sexes [15]. The laparoscopic removal has been successfully proposed in a number of retroperitoneal tumors, such as adrenal and perirenal tumors. Chens et al [18] presented the first laparoscopic resection of retroperitoneal mucinous cystadenoma of borderline malignancy in 1998. In women with PRMC-C, some authors suggest ovariectomy [11], others suggest hysterectomy [19] while some of them keep an aggressive treatment for women during the post-menopause period [20].

Due to the small amount of cases reported in the international literature, investigators could not justify any adjuvant chemotherapy protocols. The surgical procedure seems to be sufficient for the PRMC and the PRMC-BM, especially if there is not any eruption of the cystadenoma's capsule. Some authors suggest chemotherapy for the PRMC-C, based on the possible common mechanisms of histogenesis of these rare tumors with the mucinous tumors of the ovaries [15]. The small number of globally registered cases, the insufficient surveillance data and mainly the inability to understand which patients are in higher risk of recurrence, show that there is a need of an effective registration and further study of these rare tumors [12,21].

In our case, there was not any recurrence during a follow up of 24 months. Since there are no clear recommendations for surveillance concerning PRMC-BM, we proposed clinical and US examinations every six months, as well as imaging studies every year by either MRI or CT.

## Conclusion

We present a rare case of primary retroperitoneal mucinous cystadenoma of borderline malignancy in a male patient. These tumors are only twice reported in the literature. The preoperative diagnosis is difficult and laparotomy has been the standard approach for both diagnosis and treatment. Postoperative follow-up guidelines should get proposed. More similar cases must get registered, so that research defines new protocols for treatment for this domain.

## Consent

Written informed consent was obtained from the patient for the publication of this report and accompanying images. A copy of the written consent is available for review by the Editor-in-Chief of this medical journal.

## Abbreviations

US: ultrasound; CT: computed tomography; MRI: magnetic resonance imaging; PRMC: primary retroperitoneal mucinous cystadenoma; PRMC-BM: primary retroperitoneal mucinous cystadenoma-borderline malignancy; PRMC-C: primary retroperitoneal mucinous cystadenocarcinoma.

## Competing interests

The authors declare that they have no competing interests.

## Authors' contributions

FE, KS, VK, AF participated to the sequence alignment, researched sources for the references and drafted the manuscript; MS took the photoghraphs and drafted the manuscript; FE, BS, VC helped in the interpretation of the photos and helped draft the final version of the manuscript. All authors read and approved the final version of the manuscript
